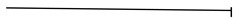# Outdoor Adventure Builds Resilient Learners for Higher Education: A Quantitative Analysis of the Active Components of Positive Change

**DOI:** 10.3390/sports7050122

**Published:** 2019-05-21

**Authors:** John F. Allan, Jim McKenna

**Affiliations:** Carnegie School of Sport, Leeds Beckett University, Leeds LS16 5LF, UK; j.mckenna@leedsbeckett.ac.uk

**Keywords:** resilience, mental health problems, higher education, outdoor adventure, multi-variate quantitative analyses, active components of positive change

## Abstract

The inability of young adults to adapt to university life has been attributed to their declining resilience. Resilience refers to any individuals’ capacity to change or modify behaviour in response to environmental hazards, so they thrive. Outdoor Adventure (OA) residential programmes have helped higher education inductees to acquire skills associated with resilience such as increased self-perception, better interpersonal relationships. However, this study addresses important gaps in existing literature by deploying a high-quality research design to examine the short-term impact of OA experiences on inductees’ resilience and to identify the active components of those experiences that best cultivate inductees’ adaptive capabilities. Multivariate analyses evaluated the efficacy of OA programming to build the resilience of over 2500 inductees. Significant positive gains were reported in the resilience of inductees attending 1-week residential OA programmes measured by an Effect size (ES) = 0.38 and 6.29% increase. Compared to students inducted at university, this represented an 8.35% greater increase in resilience (ES difference = –0.526). Camp-based experiences such as mastering new skills, developing new relationships and being female predicted heightened resilience. A defined blend of embodied, adventure-based meaningful challenges provides a template for helping university inductees to re-adjust, grow and persevere.

## 1. Introduction

Any incapacity to cope with the accumulation of stressors may cause new entrants to higher education (HE) to present worsening levels of mental health, possibly ending with dropping-out from study. Resilience represents positive adaptive behaviours necessary for healthy adjustment during periods of transition, making it a powerful potential framework for deploying strategies designed to improve student retention and achievement. 

Outdoor Adventure (OA) residential programming aims to develop a range of psycho-social characteristics consistent with enhanced resilience. These features may help HE inductees to make successful transitions into university. However, few studies based on robust empirical designs have examined the short-term impact of in-course OA experiences on inductees’ resilience. Moreover, there is a lack of understanding of the active components within OA programming which may best cultivate adaptive capabilities in young people. Beyond establishing resilience effects through OA, this study also addresses how any such effects may have resulted from specific exposures to distinct, yet common, programme experiences. This understanding could help formulate strategies which offer an appropriate balance of embodied experiences aligned to helping new students meet the real challenges of HE at a crucial time of their academic development. 

## 2. Background

The transition into university represents a pivotal, ‘in between’, period of adjustment to the interim between dependence on the family and complete independence [1]. Starting university will require students to use self-discipline to manage their academic progress alongside a possible influx of stressors, including financial uncertainty, time constraints, establishing new relationships and conforming to new social norms [2,3]. Persistent exposure to combinations of stressors in an unfamiliar environment and without established social networks may make university inductees more vulnerable to psychological and physical health problems compared to other populations [4,5,6].

Across the last decade, a global pattern of poor mental health and non-completion of first year students has been reported [6,7,8,9,10]. A number of factors have been proffered to explain this problem. Social media has been cited as detrimental to the psychological well-being of new students [11], while the reduced stigma of students reporting mental illness and increased self-referrals to university counselling services has helped to raise awareness of this issue [12]. However, the low resilience of progressively more diverse intakes of inductees, coupled with the failure of universities to adapt to meet their needs, is recognised as a key issue in the growing mental health problems and disengagement of new students [13,14,15].

Resilience refers to a person’s capacity to change or modify behaviour in direct response to environmental hazards, thrive and self-fulfil despite or even because of stressors [16]. The popularity of resilience as a construct in the psychology literature reflects a move from a deficit-based paradigm toward focus on positive qualities and their acquisition [17]. Although low resilience may not be a precursor to mental illnesses, higher levels of resilience has been synonymous with reduced vulnerability in the face of stress [18]. From this perspective, individuals are able to adapt and recover quickly from prevailing stressors (denoting bounce-back ability) and may see problems as opportunities for dynamic self-renewal (bounce-beyond ability), demonstrating resilience as a complex, non-uniform capacity for change [19].

Resilience is often acquired by confronting and overcoming adversity, enabling the development of protective mechanisms which appear important for counteracting risks [20]. These *promotive* and *protective* factors appear at the individual level, affecting self-regulation, self-esteem and determination [21], within-families and through secure attachments such as sociability and empathy [22] and through broader social and community values in education [23]. In this way, highly resourceful individuals with wide functional cognitive, social and emotional repertoires for handling the demands of HE may be considered resilient [24,25]. Despite these assertions, the theoretical and empirical analyses of the resilience of university students remains under-researched.

### 2.1. Positive Immersion within OA Experiences

Conceptually, there is an apparent fit between the stated goals of OA residential programmes and experiences that may engender *promotive* and *protective* (resilience) factors in new students [26]. Idealised, residential OA programmes represent a microcosm of the challenges facing HE inductees, including establishing peer connections, becoming familiar with new routines and managing increasing levels of cognitive and affective complexity. Previously, following participation in OA programmes, students have reported increases in resilience equating to effect sizes ranging between 0.20 (small) to 1.10 (large) compared to non-attendees [27,28,29,30,31]. Under the premise of optimising student integration into a new environment [32,33], American universities have implemented one-week Outdoor Orientation Programmes (OOPs) with variable success, reporting greater levels of physiological, emotional and social development, and improvements in academic performance [34,35,36,37].

### 2.2. OA components of Adaptive Change

A number of distinct, yet inter-connected, components of OA provide structure for the learning and transfer of such adaptive responses. These components include *the physical environment, facilitators, processes of learning* and the *learners* [38,39,40]. Responding to the *physical environment* in a variety of ways may influence subsequent resilient responses in young people. Physical interaction with nature is associated with elevated mood states [41,42,43,44], reductions in anxiety [45] and perceived restorativeness [46]. A spiritual connectedness with a greater good is signalled by a sense of awe [47,48], while being inspired by the countryside has predicted resilience outcomes in students from short-term exposure to residential OA programmes [49]. Unfamiliar OA settings foster growth by requiring youngsters to resolve the temporary disruption this causes, helping them to gain new perspectives on their usual environments [50,51]. Potential hazards in OA programmes necessitate that groups develop regard for others’ well-being. Relational resilience [52] reflects growth-fostering connections in the face of testing conditions; this is connected to individuals using the social courage needed to ask for, and give, help. 

A trusting facilitator may assist individuals to perform optimally in risk situations [53] and develop care for others [54,55]. Although there is a strong need for rationality and intentionality in adventure programming, instructors who provide authentic and immediately observable consequences for actions empower participants to self-regulate risk taking and consider the needs of others. The processes of learning in OA programming involve participants sorting and ordering meaningful information that emerges through adventurous experiences. To stimulate and accumulate meaning, OA experiences need to be intense and emotionally stimulating (therefore memorable), personally rewarding (aligned with known experiences) and relevant to everyday life (transferable) [56]. 

The nature of *learners’* age, gender and background may all have bearing on the level of engagement and nature of adaptive capabilities emanating from adventure programmes. Gender preferences for OA experiences may illustrate how differences occur and can be strengthened. Females especially valued trust activities and social interactions [57], whereas males favoured experiences related to self-determined power and dominance [58]. Although recurring characteristics of person and context emerge as predictors of resilience [59,60], attributing individuals to a particular level of resilience (low or high) will mask considerable and profound intra-personal variability. What constitutes a risk factor for one person in OA may provide opportunity for growth in another.

Notwithstanding these findings, characterising the dynamic interplay between OA residential exposure and resilient outcomes in young people is rare. Critics argue that positive behavioural outcomes emanating from OA are based on intuitive belief systems and untested assumptions rather than being derived from a broad empirical evidence base [61,62,63,64]. Methodological limitations, including over-reliance on anecdotal evidence, small sample sizes, lack of control groups, conspire to make it difficult to link short-term benefits to living and working together in unusual situations. For example, there is evidence that the first day of OA interventions tends to lower people’s feelings of well-being, self-esteem, and resilience [65]. Therefore, any subsequent increase in resilience found on the last day of the programme may not necessarily be a true reflection of increased resilience but rather a return to pre-existing levels following the dissipation of a threat or challenge [30]. A specific comparison of resilience scores between a control and intervention group on the first day of the induction would show whether this phenomenon was taking place.

In this understanding, robust empirical investigations are needed to address how outdoor experiences shape outcomes that elicit positive psychological development in HE that predict behavioural change beyond the OA context [66]. This includes evaluating interactions between individuals, genders, groups and components of programming which combine to construct psychological resilience. In this way, the appropriate type and level of experiences may be better understood and then deployed to deliver programmes which optimise healthy changes whilst avoiding maladaptive responses [67]. 

### 2.3. Research Purposes 

Current research falls short of providing robust empirical evidence of (i) short-term impacts or of establishing (ii) working mechanisms within OA programmes. Therefore, the specific purposes of this study were to:

- Establish the immediate changes to inductees’ resilience following a five-day OA residential programme;
- Compare the magnitude and direction of resilience change to similar inductees accessing comparative induction programmes at university;
- Link OA experiences and activities to the most advantageous resilience profiles by equating resilience differences with most powerful OA residential experiences and students’ perceived attributes of resilience.

## 3. Methods 

### 3.1. Participants

Sixteen first-year undergraduate courses housed within a School of Sport from a single UK university provided a purposive sample of inductees recruited over five consecutive years. This study incorporated a main ‘OA-intervention’ group who attended 40+ five-day OA residential programmes. A smaller ‘At-home comparison’ group comprised inductees from courses who chose not to access the residential experience. These students were engaged within a 5-day active programme designed to orientate them to university practices and procedures. This included practical group activities and exercises to help students interact more productively and establish familiarity with staff and the institution. 

Prior to data screening, the OA-intervention group included 2659 full-time degree students with a mean (M) age of 18.70 (±SD = 11.66) and comprised 1377 (51.8%) males and 1282 (48.2%) females. The At-home comparison group included 165 students (female, N = 89/53.9%) with a mean age of 18.62 (±SD = 1.56). After screening for incomplete questionnaires or unmatched responses, usable sample sizes included 2547 inductees (a loss of 4.2%) for the OA-intervention group and 135 inductees (a decline of 18.1%) for the At-home comparison group. In both groups, participants were predominately white (96.8%), making this sample highly compatible with a normative HE population of inductees. Across the UK, HE sectors in 2010/11, over two-thirds of all full-time first-degree students were either 18 or 19 years of age on entry, 45.8% were male and 84.7% were white [68].

This ‘widening participation’ university annually enrolled approximately 6000 new full-time degree inductees. More than 9 of 10 students were state educated and over one-third came from socio-economic groups 4–7 such as lower supervisory and technical, routine and semi-routine occupations. The UK Universities and Colleges Admissions Service (UCAS) tariff for admission into the School of Sport equates to 80 points. Advanced-level (A-level) qualifications are afforded an incremental tariff for entry into HE; grade E = 16 points; A* = 56 points. All participants gave informed consent for their involvement.

### 3.2. Design and Measures

Following institutional ethical approval, this study comprised three stages of investigation which matched the specific research purposes of the study. All analyses were conducted using the Statistics Package for the Social Sciences (SPSS) Version 24 [69].

#### 3.2.1. Stage 1: Immediate Impact of OA on Inductees’ Resilience 

Inductees from both sample groups completed the self-reported Connor–Davidson Resilience Scale (CD-RISC) [18] immediately before and directly following their respective five-day OA residential/university-based induction programmes. This scale is suitable for use with older adolescents in educational contexts [70]. The CD-RISC generates Total Resilience (0–100) and five contributory subscale scores of (i) Competence (0–32), (ii) Trust (0–28), (iii) Change (0–20), (iv) Control (0–12) and (v) Spirituality (0–8). CD-RISC comprises 25 phrases such as ‘I adapted to change’ with scoring ranging from 0 (Not at all true) to 4 (True nearly all the time); higher scores reflect greater resilience. 

CD-RISC TR internal consistency (Cronbach’s α) was 0.92 and for the subscales as follows: Competence (0.86), Trust (0.77), Change (0.75), Control (0.65), Spirituality (0.60). Test–retest reliability demonstrated a high level of agreement with an intra-class correlation coefficient of 0.87. Construct validity was confirmed with high convergent correlations (r = 0.83, *p* < 000.1) with the Kobasa Hardiness Scale [71], and appropriately negative discriminant correlations (r = 0.34, *p* = 11) with the Arizona Sexual Experience Scale (ASEX) [72].

A progressive series of statistical tests established the significance, magnitude and direction of change in the OA-intervention groups’ resilience following the OA residential. Paired t tests, a one-way ANOVA, a 2 × 2 mixed-design ANOVA and Multivariate Analyses of Variance (MANOVA) established differences in CD-RISC Total Resilience difference (TRdiff) and the five resilience subscales by gender and annual cohort. The magnitude and direction of changes were represented by Cohen’s *d* effect size (ES) and percentage differences. A general convention is to interpret the ESs of approximately 0.20 to be ‘small’ in magnitude, those of 0.50 to be ‘moderate’ and those larger than 0.80 to be ‘high’ [73]. To provide a further sensitised evaluation of the responsiveness of subgroups of inductees to the OA intervention, quartile ranges were constructed signifying differences in Total Resilience and subscales ranging from ‘negative differences’ to ‘high positive differences’. Chi-square tests established any significant differences between the frequency of observations in the categories.

#### 3.2.2. Stage 2: Comparative Analyses of Resilience Change

To test the efficacy of the OA residential programmes, CD-RISC scores of the OA intervention group were compared to the At-home comparison group. Comparative analyses were undertaken using independent t tests, a 2 × 2 mixed-design ANOVA, MANOVA and effect sizes.

#### 3.2.3. Stage 3: Powerful Components/Personal Experiences of OA Programming

OA within the residential week involved participants’ taking personal risks through progressive exposure to a range of outdoor activities designed to develop adaptive capabilities. These activities included team challenges, educational visits, rock climbing and abseiling, ghyll scrambling, bivouacking, mountain-walking, canoeing and kayaking. Although similar activities and practices were delivered across residential venues, there were course specific educational objectives embedded into specific programmes. Programmes were delivered across varied outdoor locations (The English Lake District, Yorkshire Dales and North Wales).

Immediately following completion of the OA programme, two bespoke instruments captured OA-intervention inductees’ self-perceptions of their (i) level of immersion within OA components of the programme and (ii) competencies acquired associated with adaptive functioning across the duration of the programme. 

A 19-item *Camp Rating Scale* (CRS) measured inductees’ immersion within the programme. Items were formulated from previous School of Sport student reviews of OA residential programmes consistently identified as important for transition. A graduated five-point Likert scale enabled responses ranging from ‘Never’ (=1), indicating no engagement in camp-related activities, to the highest rating of ‘Through most days’ (=5). The internal reliability of the scale was identified as ‘acceptable’ (α 0.72). A 15-item *Perceived Competencies Scale* (PCS) enabled inductees to attribute changes to recognised adaptive behaviours, ranging from ‘Much worse’ (=1) to ‘A lot better’ (=5). Items comprised protective and promotive factors which could underpin changes in resilience. The internal reliability of the scale was identified as ‘excellent’ (α 0.88).

To identify factors from the two bespoke scales which predicted differences in resilience, a stepwise multiple linear regression analysis was undertaken. Multiple linear regression aids prediction by determining the degree to which a combined set of predictors, which may be measured on continuous (interval or ratio) and categorical (dichotomous) scales, predict a criterion. Used with large sample sizes, stepwise regression progressively selected the order in which predictors were established. A stepwise binary logistic regression analysis was also performed to identify those items on the rating scales most likely to predict inductees belonging to quartile groups of resilience difference (ranging from ‘negative differences’ to ‘high positive differences’. This model systematically identifies significant variables to predict the change in odds that observations belong to one of two groups. Scores for items on each scale were used to predict the likelihood of inductees belonging to the optimum ‘high positive’ resilience difference group, using the other quartile ranges as comparisons. This analysis helps to establish the most powerful active ingredients within the OA programmes alongside the personal attributes that generate behavioural change.

## 4. Results

### 4.1. Stage 1: Changes to CD-RISC Total Resilience (TR) and Subscales

Table 1 shows inductees’ mean baseline and post-intervention scores for CD-RISC Total Resilience (TR) and subscales. Paired t tests revealed significant positive differences in TR and all subscales, showing a positive intervention effect on all resilience subscales. Effect sizes (ES) for TR and subscales were between ‘small’ and ‘moderate’ with the exception of Spirituality, where the ES was less than ‘small’. The ES of 0.38 (range 0.13 to 0.86) for TR constituted a 6.29% increase. The largest percentage increase in subscales was seen in Control (8.66%). Indicating that females acquired a wider array of powerful, positive influences on behaviour than their male counterparts, females displayed the highest ES in TR, Competence, Trust, Change and Spirituality. Males recorded the largest ES in Control. 

There were no significant gender differences in mean Total Resilience difference (TRdiff). Only the subscale of Change in females (M = 1.08, ±SD = 2.75) compared to males (M = 0.76, ±SD = 2.93) showed a significant mean difference F (1, 2547) = 8.35, *p* < 0.00. A 2 × 2 × 2 mixed-design ANOVA for baseline and post-intervention TR by annual cohort and gender revealed significant mean differences across baseline and post-intervention TR scores F (1, 2537) = 579.21, *p* < 0.001. TRdiffs were significantly different between annual cohorts F (4, 2537) = 53.59, *p* < 0.001, by year and gender F (4, 2537) = 3.30, *p* < 0.05. 

To provide a sensitised evaluation of resilience difference, re-classified quartile ranges of TRdiff and subscales were developed. Table 2 quartiles reflect categories from ‘Negative’ differences (−31 to –2) to ‘High Positive’ differences (9 to 47). Most inductees were located in the positive difference quartile groups for TR and subscales. Over three-quarters of all participants recorded scores of −1 or above in TR from a possible range of −37 to 47. Over 85% of inductees reported positive changes in Control. More males achieved negative difference categories for TR and all subscales, with the exception of Control. Change was significantly associated with differences between observed and expected frequencies in the quartile categories χ 2 (1) = 15.52, *p* < 0.01.

### 4.2. Stage 2: OA-Intervention Group versus At-Home Comparison Group

The At-home comparison group displayed no significant changes to their resilience and subscales following the induction week. Their TR and subscales showed negative ESs and percentage decreases. TR reduced by 2.06%. The subscales mostly contributing to the decline in resilience were Competence (−3.19%) and Trust (−1.90%). A comparison was made of baseline scores for TR and subscales between the OA-intervention and At-home comparison group. Although there were no significant differences between their scores, the OA-intervention group scored higher across all measures. Table 3 highlights post-intervention mean differences (significance, magnitude and direction of TR and subscales) between the OA-intervention and At-home comparison group. The majority of mean differences were significant at the 0.01 level for the OA group. Effect sizes were ‘high’ (with the exception of Spirit), reflecting that the OA-intervention group outperformed the At-home comparison group in reporting positive changes as a function of time (pre/post). Most importantly, the effect size for TRdiff between the At-home comparison and OA-intervention group was −0.526, (OA group ES = 0.38; comparison group ES = −0.15) which represented difference of 8.35% in TR. The subscale that accounted for the greatest difference in ES between the groups was Competence (ES = −0.59, 9.63% difference).

**Figure 1:**
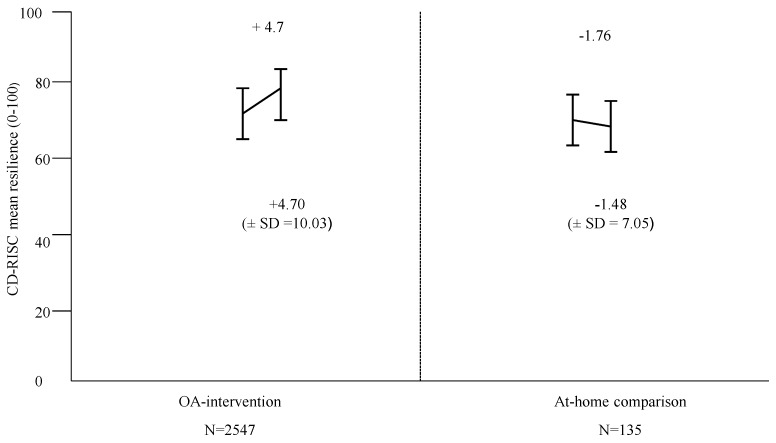
Mean difference in CD-RISC Total resilience difference by condition. MANOVA F (1, 2678) = 46.42, *p* < 0.001.

**Figure 2:**
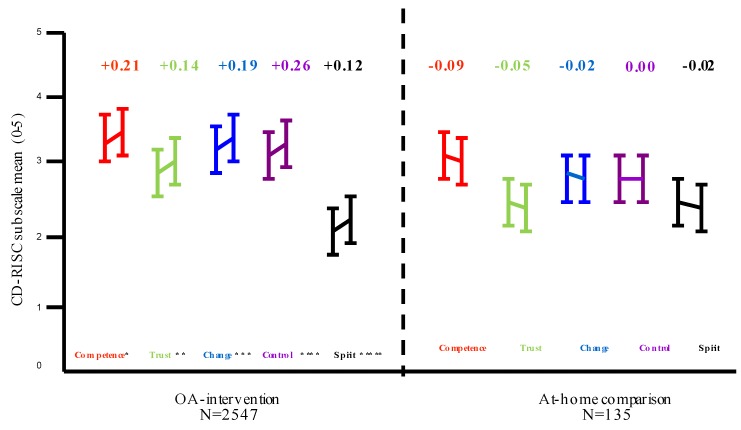
Mean difference in CD-RISC subscales by condition. MANOVA ALL F (1, 2678), * = 49.55, *p* < 0.001, ** = 14.99, *p* < 0.001, *** = 17.15, *p* < 0.001, *** = 39.96, *p* < 0.001, **** = 5.21, *p* < 0.05.

Given that the descriptive analyses suggested differences to changes in resilience between the OA- intervention and At-home comparison group, a 2 × 2 mixed-design ANOVA compared the differences in TR between the two groups by gender. OA-intervention group vs. At-home comparison group and gender were the between-subjects’ factors and time (resilience measurement day 1 vs. day 5) comprised the within-subjects’ factors. A significant main effect of TR measurement was observed. Total mean resilience was significantly higher post OA (M = 74.97) compared to baseline (M = 73.31), F (1, 2678) = 14.02, *p* < 0.001. An interaction between time and group indicated a significantly greater change in TR for the OA-intervention group F (1, 2678) = 46.43, *p* < 0.001. There was no significant interaction between time and gender F (1, 2678) = 0.426, *p* > 0.05, and time × condition × gender F (1, 2678) = 0.020, *p* > 0.05. A MANOVA revealed significant mean differences in TRdiff and for all subscales between the OA-intervention and At-home comparison group following one-week interventions (Figure 1 and Figure 2). There were no significant differences by gender or condition × gender.

### 4.3. Stage 3: Influential Programme Experiences: Camp Rating Scale (CRS) and Perceived Competencies Scale (PCS)

Table 4 depicts OA-intervention inductees’ mean level of perceived engagement within 19 OA residential activities from the *Camp Rating Scale* (CRS). Ratings indicated that individuals were actively engaged with 15 of the 19 activities ‘Every day’. Students were able to consistently engage with others, become self-reliant and skilled in a broad range of areas while hardly ever feeling homesick. The lowest categories of engagement included ‘Being able to choose the OA’ and ‘Being able to self-cater’ reflects that most programmes contained a standard programme of activities and were fully catered.

Table 5 illustrates inductees’ perceived level of change to components of resilient behaviour from the *Perceived Competencies Scale* (PCS). Positive improvements were reported by inductees in all 15 facets of behaviour related to hallmarks of resilience such as social connectedness, cognitive and emotional competence. The greatest perceived changes were realised in developing relationships and coping with present uncertainty.

Multiple stepwise linear regressions revealed that seven OA programme experiences and competencies predicted inductees’ heightened resilience. Three CRS items significantly influenced TRdiff, ‘Learned and mastered new skills’ (which was the most powerful, βeta = 0.082, t = 3.800, *p* ≤ 0.01), ‘Could act in an independent way’ and ‘Getting along well with people in my group’. Four items from the PCS scale included ‘My social relationships now’ (the most influential, βeta = 0.108, t = 4.184, *p* ≤ 0.01), ‘My level of optimism now’, ‘My motivation to study now’ and ‘Manage life’s ups and downs now’. Gender was not a predictive influence on TRdiff. 

Multiple stepwise binary logistic regression highlighted items on the CRS and PCS which most likely predicted inductees’ membership of the ‘High Positive Difference’ quartile group. All multiple stepwise binary logistic regression (χ2 (1, 1089) *p* < 0.05) demonstrated that being female (OR = 1.351, 95% C.I. 1.060 to 1.721) alongside three CRS items predicted membership of the ‘High Positive’ resilience difference quartile group (26.0% of inductees) compared to the ‘Negative’ resilience difference group (24.1%). The CRS items were ‘Learned and mastered new skills’ (OR = 1.221, 95% C.I. 1.053 to 1.415), ‘Free to make own decisions’ (OR = 1.154, 95% C.I. 1.013 to 1.314) and ‘Left behind usual tobacco, drug or alcohol behaviours’ (OR = 1.145, C.I. 1.018 to 1.289). In this model, females were 35% more likely to be in the ‘High Positive’ group compared to males; for every unit increase in learning new skills, students were 22% more likely to be in the group with the highest positive resilience difference. The PCS items of ‘My mental strength now’ (OR = 1.426, 95% C.I. 1.135 to 1.759) and ‘My social relationships now’ (OR = 1.418, 95% C.I. 1.123 to 1.791) predicted membership of the ‘High Positive’ resilience difference group by 42 and 41% respectively. These analyses were confirmed by tests of appropriate goodness of fit, sample size, multi-co-linearity, classification accuracy, and cross sample validation.

## 5. Discussion

This research has established three main findings. First, significant positive gains were reported in the resilience (ES = 0.38, 6.29% increase) of considerable numbers of inductees within and across five years, representing 40+ OA residential programmes. Second, on average, residential OA inductees achieved an 8.35% greater increase in resilience, compared to inductees who reported negative outcomes following university-based induction programmes (ES difference = −0.526). Third, frequent immersion of OA inductees within key components of OA programming and increases in perceived competencies predicted their heightened resilience. 

These findings confirmed the positive impact of OA residential programming on the adaptive capabilities of new students. These data not only provide powerful evidence for OA developing immediate improvements in inductees’ resilience but also identified the type of experiences and degree of exposure which generated these changes. The results established that these acquired skills and knowledge predicted inductees’ resilience and linked these experiences and competencies to specific clusters of students. A variety of emotional responses reflected the inductees’ adjustments to the challenges of these OA programmes. Nonetheless, students perceived developing a more controlled presence of mind and adoption of behaviours that helped manage any accumulation of stressors that might otherwise have triggered adverse emotional responses. Embodied, meaningful challenges which required inductees to realign their perceived capabilities may help to normalise difficulties that all learners face in HE and enable them to re-adjust, grow and persevere in their academic studies.

### 5.1. Short-Term Impact of OA on Inductees’ Resilience

OA residential programmes initiated significant heightened resilience. Seven of 10 OA participants achieved positive differences in resilience and subscales. Over 85% of inductees reported positive changes in their ability to exert control over stressors. While the scale of changes to resilience differed between annual cohorts, positive gains were reported by inductees within and across all years. The magnitude and direction of changes (ES) were equivalent to the ESs of OA programming which were educationally significant (0.31 and 0.50) and represented therapeutic value for young people (range from 0.30 to 0.50) [43,44]. The findings were consistent with adaptive skill sets resulting from policy initiatives [65] and in-line with the ESs of much smaller previous similar studies [27,28,29,30]. These findings provide a powerful justification for using OA residential programming for developing immediate increases in the resilience of HE inductees. 

### 5.2. Comparison Group

This study provides a strong contrast group, based on an induction programme conducted ‘At-home’, on campus, within the home university. The ESs for resilience and subscales were positive and ‘moderate’ for OA inductees, whereas non-OA attendees reported negative ESs and percentage decreases. Differences in the resilience outcomes were almost 9% greater for the OA-intervention group than in the At-home comparison. These findings support previous studies wherein the greatest improvement in the adaptive capabilities were developed through active OA induction programmes compared to traditional induction practices [29,31]. 

The resilience subscale that accounted for the greatest ES differences between the two groups was perceived Competence. Furthermore, within-group analyses identified that OA-intervention students’ higher resilience was progressively and incrementally associated with how frequently they learned new skills. OA residential programmes typically require participants to continually re-evaluate their capabilities through problem solving in small groups within an authentic setting where consequences for actions are realistic. Although the OA condition may have been uncomfortable for some students (reflected in their negative responses), differences between the OA and At-home conditions highlight the potential problems of relying on existing locally based provision. This is unlikely to replicate the naturally emergent, experiential forms of learning which accompanies OA-based resilience building. 

### 5.3. Components of OA Residential Programming

Resilient responses to *physical environments* in OA have ranged from participants’ resolving disruption to their well-being, feeling psychologically restored and developing a spiritual connectedness with nature [41,42,46,48]. Although inductees’ spirituality was unaffected by OA programming, resilience was most evident through being free to test competencies, being required to get along with others, leaving behind old habits and dealing with uncertainty. 

Regular social interactions between inductees and *facilitators* underpinned students’ positive adaptive responses. Relational qualities associated with resilience, namely asking for help, establishing support networks, feeling connected to a broader community, all help to buffer the impact of stress on new university students. Predictive models in the current study highlighted the importance of students establishing social connections and becoming more self-determined for building their resilience. Refining the behaviours that develop autonomy may liberate participants to self-regulate and develop concern for others; these are important qualities for new students to deal with difficulties encountered at university [4,6,7,8].

Deploying *processing* strategies within OA programming, such as structured reflection, enables participants to internalise the meaning of their experiences and consolidates learning across contexts [39,40]. The current data show that specific behaviours predicted both inductees’ resilience (learning new skills, mental strength, sociability, freedom of choice) and changes in the sub-domains of the CD-RISC. Given that a perennial challenge in OA programming is to transfer any newly acquired skills of participants with challenges faced in everyday life, our findings provide a template for behaviours aligned with ‘items that create the sub-domains on the CD-RISC. These convert into teachable behaviours, pedagogies and practices, many of which have proven worth for promoting retention and achievement in HE [34]. 

Individual challenges to perceptions of capabilities ensure *learners* will display a variety of adaptive responses in OA contexts. Data indicated substantial variability within and between annual cohorts and sub-groups of inductees. For example, over one-fifth of new students reported decreases in their resilience and distinctive gender-based patterns of functioning were established [58]. Female inductees acquired a more powerful, wider repertoire of adaptive functioning than males; this emerged through experiences involving the learning new skills, building social relationships, forgiving personal shortcomings, and coping with uncertainty. In contrast, males preferred having the freedom to make decisions and the opportunity to solve their own problems. Previously, females reported higher levels of socialisation in OA programming within HE [29], while males placed a higher value on autonomy [58]. Findings from the current study suggest a more nuanced, tailored approach is more likely to meet the specific transitional needs of inductees. Further, gendered responses can help to construct better OA-based inductions. While it is possible that negative resilient outcomes reflected an advantageous process of reassessment, it is equally important that responses to any programme are not used to stigmatise some groups. More males than females featured in the high resilience difference categories for Competence and for Control. 

### 5.4. Strengths and Limitations

The current research was based on clear chains of inferential reasoning supported and justified by rigorous, objective empirical practices across five annual cohorts. This is the largest study of its kind and, combined with the use of a comparison sample, it is both statistically powerful and internally valid. The similarity of resilience scores on the first day of the induction between the groups provide confidence that positive resilience outcomes were due to elements of OA programming. Anticipatory lower resilience reported immediately prior to OA programmes have brought doubt on the validity of increased outcomes in previous studies [29,65]. Further, strength arises from showing that the high completion rates of a comprehensive range of valid and reliable measures were sensitive to inductees’ outcomes, predicting both the direction and magnitude of change. 

Nonetheless, a number of methodological caveats affect the findings. First, this study was restricted to students within a single UK university, limiting generalisability across the HE sector. The pre-/post- time series design remedied the pitfalls of cross-sectional techniques. However, non-longitudinal analyses made it difficult to gauge the degree of transfer to other settings. As with any questionnaires requiring self-evaluation, establishing differences between pre-test and post-test scores within OA programmes may have been affected by the timing of measurement. Pre-group measures could reflect participants’ anticipation of confronting something new, making them lower-than normal estimates of personal capability. Equally, measures captured immediately following the programme may detect ‘post-group’ euphoria. Remedied, these issues may reduce the magnitude of overall programme effects. Allowing for the strengths associated with a ‘At-home’ comparison group, the relatively small sample may be unrepresentative. Further, this study focused specifically on the OA-intervention groups’ programme of activities and not aspects of the university-based induction programme. 

## 6. Summary and Conclusions

The aim of this study was not to portray resilience as a panacea for ‘fixing’ all aspects of adaptive functioning in new students. This study was designed to explore how enhanced resilience is predicted by manageable combinations of enabling processes and programme approaches. It also answers calls for empirically robust investigations into the impacts of OA programmes, and for HE to deliver engaging, induction practices which can develop a buffering capacity of first-year stressors.

This study provides powerful evidence that resilience was derived from actively confronting challenges. This indicated a purposeful compatibility between inductees’ needs and the dynamics of known components of OA programming. Crucially, sub-domains of resilience, such as the capacity to make friends, solve problems and take control were all heightened through, and predicted by, frequent exposure to distinct OA experiences and practices. This substantiates the use of appropriately challenging pedagogical approaches in HE; none appeared to worsen inductees’ perceived fragility. Moreover, these authentic learning experiences have helped inductees to appreciate the value of effort-driven behaviours and to draw as much learning from their unsuccessful activities as they do from success. This experience has strong possibilities for helping inductees to become self-reliant students with critical awareness. 

Given the complexity of risk and resilience transactions in young people, there may be problems in promoting lists of universal assets arising from OA programming across groups, contexts and time. In the current study, evidence of negative emotional reactions to challenges, of cohort-specific and of gendered responses underlines that both universal ‘guidelines’ can be blended with individualising influences to optimise adaptive functioning. As an evidence-based approach, measures from this research could be used in a longitudinal study to investigate the sustainability of OA programming for students in the early experiences of HE. Further, future studies should ascertain how resilience reduces students’ stress and correlates with learning measured through educational outcomes. Nonetheless, the embodied learning experiences reported offer powerful evidence for HE providers. The predictive programme components may be useful for planning to influence comparable inductee groups. This evidence constitutes a broad set of readily available assets and resources to a community of practice intent on building individual and collective agency to protect against the potential for drop-out among new students. 

## Figures and Tables

**Table 1:** Baseline and post-intervention mean Connor–Davidson (CD-RISC) Total Resilience (TR) and subscale differences of Outdoor Adventure (OA)-intervention inductees by gender for all annual cohorts.

	Means (±SD) (n)														
	Baseline			Post			Differences *			Cohen’s d Effect Size (ES) †			% Difference (+)		
Variable (Range)	Males (1309)	Females (1238)	All (2547)	Males (1309)	Females (1238)	All (2547)	Males	Females	All	Males	Females	All	Males	Females	All
CD-RISC (0–100)	74.64 (12.38)	74.93 (12.84)	74.77 (12.59)	78.98 (11.90)	79.98 (12.23)	79.47 (12.07)	t(1308) = 15.22	t(1237) = 18.27	t(2546) = 23.55	0.36	0.40	0.38	5.81	6.74	6.29
Competence (0–4)	3.26 (0.55)	3.25 (0.55)	3.26 (0.55)	3.47 (0.50)	3.47 (0.51)	3.47 (0.51)	t(1308) = 15.47	t(1237) = 16.87	t(2546) = 22.78	0.40	0.41	0.40	6.44	6.76	6.44
Trust (0–4)	2.84 (0.55)	2.83 (0.56)	2.84 (0.56)	2.97 (0.58)	3.00 (0.58)	2.98 (0.58)	t(1308) = 7.83	t(1237) = 11.61	t(2546) = 13.60	0.23	0.30	0.25	4.57	6.00	4.93
Change (0–4)	3.12 (0.60)	3.12 (0.63)	3.12 (0.61)	3.27 (0.57)	3.34 (0.56)	3.31 (0.57)	t(1308) = 9.44	t(1237) = 13.92	t(2546) = 16.33	0.26	0.37	0.32	4.80	7.05	6.09
Control (0–4)	3.00 (0.64)	3.02 (0.67)	3.00 (0.66)	3.27 (0.62)	3.26 (0.65)	3.26 (0.64)	t(1308) = 16.07	t(1237) = 14.08	t(2546) = 21.34	0.43	0.36	0.40	9.00	7.95	8.66
Spirit (0–4)	2.00 (0.97)	2.21 (0.91)	2.10 (0.94)	2.11 (1.02)	2.33 (0.94)	2.22 (0.99)	t(1308) = 5.27	t(1237) = 6.60	t(2546) = 8.34	0.11	0.13	0.12	5.50	5.43	5.71

* All *p* < 0.01; † Effect size (ES) 0.2—small, 0.5—moderate, 0.8—large.

**Table 2:** CD-RISC Total Resilience (TR) and subscale baseline and post-intervention difference ranges for OA intervention group by gender.

	Categories of Change for Total Resilience															
Variable	Negative %				Small Negative to Positive %				Small Positive %				High Positive %			
	Range Min to Max	M %	F %	All %	Range Min to Max	M %	F %	All %	Range Min to Max	M %	F %	All %	Range Min to Max	M %	F %	All %
CD-RISC	−31 to −2	25.8	22.4	24.1	−1 to 2	22.4	22.2	22.3	3 to 8	27.3	27.9	27.6	9 to 47	24.6	27.5	26.0
Competence	−12 to −2	16.7	13.6	15.2	−1 to 0	25.9	27.3	26.6	1 to 3	29.2	33.1	31.1	4 to 18	28.2	26.1	27.1
Trust	−15 to −2	26.1	21.1	23.6	−1 to 0	22.7	24.0	23.4	1 to 2	21.0	21.6	21.3	3 to 15	30.2	33.3	31.7
Change *	−14 to −2	16.8	12.4	14.7	−1 to 0	35.4	33.3	34.4	1 to 2	27.0	32.1	29.5	3 to 14	20.7	22.2	21.5
Control	−5 to −2	20.1	22.9	21.5	0 to 1	49.9	47.6	48.9	2 to 3	23.0	22.3	22.6	4 to 8	7.0	6.8	6.9
Spirituality	−6 to −2	10.1	8.1	9.1	−1 to 0	51.3	53.8	52.5	1 to 2	31.5	31.5	31.5	3 to 6	7.1	6.6	6.9

* Pearson’s chi-square 2 (1) = 15.52, *p* < 0.01.

**Table 3:** Post-intervention mean differences for CD-RISC Total Resilience (TR) for OA-intervention and At-home comparison group by gender.

	Post Mean Differences (±SD) (n)														
	OA-Intervention Group			At-Home Comparison Group			Differences †			% Difference (+)			Cohen’s d Effect Size (ES) *		
Variable (Range)	Male (1309)	Female (1238)	All (2547)	Male (61)	Female (74)	All (135)	Males	Females	All	Males	Female	All	Male	Female	All
CD-RISC (0–100)	78.98 (11.90)	79.98 (12.23)	79.47 (12.07)	70.10 (9.46)	70.56 (10.58)	70.35 (10.06)	t(1368) = 5.68	t(1310) = 6.45	t(2680) = 8.55	12.66	13.35	12.96	0.83	0.82	0.82
Competence (0–4)	3.47 (0.50)	3.47 (0.51)	3.47 (0.51)	3.00 (0.53)	3.04 (0.48)	3.03 (0.49)	t(1368) = 7.05	t(1310) = 6.94	t(2680) = 9.88	15.66	14.14	14.52	0.91	0.87	0.88
Trust (0–4)	2.97 (0.58)	3.00 (0.58)	2.98 (0.58)	2.54 (0.43)	2.60 (0.48)	2.57 (0.46)	t(1368) = 5.31	t(1310) = 5.87	t(2680) = 7.87	16.93	18.11	15.95	0.86	0.75	0.78
Change (0–4)	3.27 (0.57)	3.34 (0.56)	3.31 (0.57)	2.94 (0.43)	2.94 (0.54)	2.94 (0.49)	t(1368) = 4.47	t(1310) = 6.12	t(2680) = 7.45	11.22	13.60	12.58	0.66	0.73	0.70
Control (0–4)	3.27 (0.62)	3.26 (0.65)	3.26 (0.64)	2.79 (0.61)	2.82 (0.58)	2.81 (0.60)	t(1368) = 5.80	t(1310) = 5.64	t(2680) = 8.10	17.20	15.60	16.01	0.78	0.72	0.73
Spirit (0–4)	2.11 (1.02)	2.33 (0.94)	2.22 (0.99)	2.36 (1.05)	2.46 (0.98)	2.42 (1.01)	NS	NS	t(2680) = 2.25	−10.59	−5.28	−8.26	−0.24	−0.13	−0.20

* Effect size (ES) 0.2—small, 0.5—moderate, 0.8—large, † *p* < 0.01.

**Table 4:** *Camp Rating Scale* (CRS), Mean (SD) responses.

Variable	Range
	Never Through Most Days
	1 2 3 4 5
1. With people of my own age	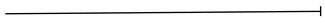
2. Got on well with people in my group	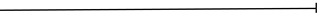
3. Took part in adventure activities	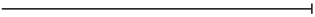
4. Able to laugh at myself	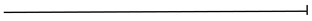
5. Learned and mastered new skills	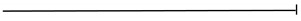
6. Motivated by the activities I did	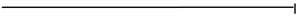
7. Solved my own problems	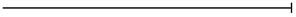
8. Took responsibility for things	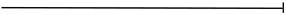
9. Took part in formal team-building exercises	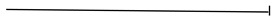
10. Good connections with residential staff	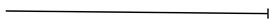
11. Left behind usual unhealthy habits	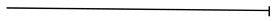
12. Could act in an independent way	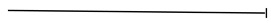
13. Enjoyed social and academic activities	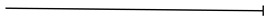
14. Experienced camp leaders	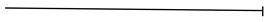
15. Free to make my own decisions	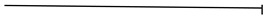
16. Inspired by the countryside	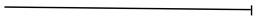
17. Able to choose activities I did	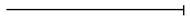
18. Cooked for myself and the group	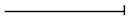
19. Felt homesick	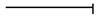

**Table 5:** *Perceived Competencies Scale* (PCS) Mean (SD) responses.

Variable	Range
	Never Through Most Days
	1 2 3 4 5
1. My social relationships now	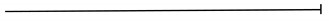
2. My coping with unfamiliar events now	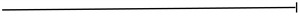
3. My personal growth now	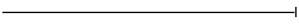
4. My mental strength now	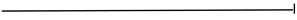
5. My level of optimism now	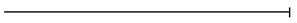
6. My resourcefulness now	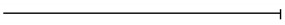
7. How well I know myself now	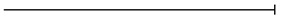
8. My creativity now	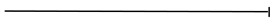
9. My ability to predict how others will react	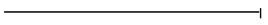
10. Forgive others shortcomings now	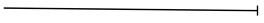
11. My motivation to study now	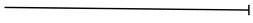
12. My connection to the world now	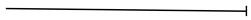
13. Manage life’s ups and downs now	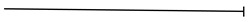
14. Forgive own shortcomings now	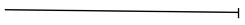
15. My level of hostility now